# Brain Targeted AAV1-GALC Gene Therapy Reduces Psychosine and Extends Lifespan in a Mouse Model of Krabbe Disease

**DOI:** 10.3390/genes14081517

**Published:** 2023-07-25

**Authors:** Aimee R. Herdt, Hui Peng, Dennis W. Dickson, Todd E. Golde, Elizabeth A. Eckman, Chris W. Lee

**Affiliations:** 1 Biomedical Research Institute of New Jersey, Cedar Knolls, NJ 07927, USAlizeckman@brinj.org (E.A.E.); 2 MidAtlantic Neonatology Associates (MANA), Morristown, NJ 07960, USA; 3 Atlantic Health System, Morristown, NJ 07960, USA; 4 Department of Neuroscience, Mayo Clinic, Jacksonville, FL 32224, USA; 5 Department of Pharmacology and Chemical Biology, Emory University, Atlanta, GA 30322, USA; 6 Department of Neurology, Emory University, Atlanta, GA 30322, USA; 7 Emory Center for Neurodegenerative Disease, Emory University, Atlanta, GA 30322, USA

**Keywords:** Krabbe disease, galactosylceramidase, psychosine, twitcher, adeno-associated virus serotype 1, gene therapy

## Abstract

Krabbe disease (KD) is a progressive and devasting neurological disorder that leads to the toxic accumulation of psychosine in the white matter of the central nervous system (CNS). The condition is inherited via biallelic, loss-of-function mutations in the galactosylceramidase (*GALC*) gene. To rescue *GALC* gene function in the CNS of the twitcher mouse model of KD, an adeno-associated virus serotype 1 vector expressing murine *GALC* under control of a chicken β-actin promoter (AAV1-GALC) was administered to newborn mice by unilateral intracerebroventricular injection. AAV1-GALC treatment significantly improved body weight gain and survival of the twitcher mice (*n* = 8) when compared with untreated controls (*n* = 5). The maximum weight gain after postnatal day 10 was significantly increased from 81% to 217%. The median lifespan was extended from 43 days to 78 days (range: 74–88 days) in the AAV1-GALC-treated group. Widespread expression of GALC protein and alleviation of KD neuropathology were detected in the CNS of the treated mice when examined at the moribund stage. Functionally, elevated levels of psychosine were completely normalized in the forebrain region of the treated mice. In the posterior region, which includes the mid- and the hindbrain, psychosine was reduced by an average of 77% (range: 53–93%) compared to the controls. Notably, psychosine levels in this region were inversely correlated with body weight and lifespan of AAV1-GALC-treated mice, suggesting that the degree of viral transduction of posterior brain regions following ventricular injection determined treatment efficacy on growth and survivability, respectively. Overall, our results suggest that viral vector delivery via the cerebroventricular system can partially correct psychosine accumulation in brain that leads to slower disease progression in KD.

## 1. Introduction

Krabbe disease (KD) (OMIM 245200) is a lysosomal storage disorder (LSD) characterized by demyelination of the central nervous system (CNS) [1]. It is a monogenetic, autosomal recessive disease caused by deficiency in galactosylceramidase (GALC, EC 3.2.1.46) enzymatic activity [2]. GALC activity is predominantly localized in lysosomes, where it is essential for normal catabolism of galactolipids, including psychosine and a major myelin component, galactosylceramide. GALC deficiency results in progressive accumulation of psychosine, which is cytotoxic to oligodendrocytes and Schwann cells [3,4]. Loss of these myelin-forming cells causes demyelination and neurodegeneration in both the CNS and the peripheral nervous system (PNS) during early developmental stages [5]. Clinically, the onset of infantile KD occurs between three and six months of age. Initial symptoms include spasticity, irritability, and hypersensitivity to external stimuli, followed by opisthotonic posturing, visual failure, hypertonic fits, and loss of tendon reflexes; death typically occurs before two years of age. Treatment options for KD are mainly palliative, with the exception of hematopoietic stem cell transplantation (HSCT) which can slow disease progression in infantile KD patients when treated at a pre-symptomatic stage [6,7] or in late-onset KD patients treated during an early clinical stage [8]. 

The twitcher mouse is the most widely used animal model to study pathology and experimental therapeutics in KD [9,10]. It carries a homozygous, nonsense mutation (p.W339X) on the *GALC* gene that abolishes GALC protein expression through a nonsense-mediated mRNA decay mechanism [11]. Complete loss of GALC function leads to abnormal accumulation of psychosine, particularly in nervous tissues [12,13]. The most common variant observed in human infantile KD is a 30 kb deletion from exon 11 through exon 17, which also causes a complete loss of GALC protein [14]. Thus, the twitcher mouse presents an authentic genetic and biochemical disease model for infantile KD. Clinically, twitcher mice have a failure to thrive phenotype, characterized by slow weight gain through postnatal day (PND) 32, followed by precipitous weight loss until death around PND 40 [15]. A decrease in motor function becomes apparent at PND 15, and the motor function rapidly declines after PND 20 [16]. Pathologically, globoid cells are present in twitcher mice and increase in number in the white matter of the CNS and the PNS beginning at PNDs 10−15 [17]. Neuroinflammation including microglial activation and astrogliosis are also prominent and can be detected at two to three weeks [18]. Demyelination in the CNS and the PNS can be detected after PND 21, and its progression is associated with neurological symptoms such as tremor and spasticity [19,20]. Recently, CD8-positive cytotoxic T lymphocytes were also found in the CNS of twitcher mice and implicated to play a role in initiation of clinical symptoms in the model [21].

The use of adeno-associated viral (AAV) vectors has emerged as a strategy to achieve widespread in vivo gene replacement in the CNS and the PNS. A growing number of gene therapy studies using AAV vectors have yielded promising results in preclinical applications, paving the way for phase I and II clinical trials using AAV to treat the CNS-affecting LSDs (reviewed in [22,23]). One clinical trial to treat KD patients combines hematopoietic stem cell transplantation and intravenous AAV serotype rh10-*GALC* gene therapy (ID: NCT04693598). The nonblinded, non-randomized trial is ongoing with plans to enroll six KD infants. Another trial was planned to study AAV serotype hu68-GALC administered into cisterna magna (NCT04771416). Unfortunately, the clinical trial was halted by the sponsor in early 2023 due to product prioritization. Several different AAV serotypes have been considered for gene therapy studies; while AAV serotype 1 (AAV1) is one of the most common viral vectors tested, not much information is available on its application in KD. Here, we report on the results of a preclinical gene replacement study with AAV1-GALC in the twitcher mouse model of KD, including a description of GALC protein distribution and psychosine levels in different brain regions, which may provide useful information regarding treatment efficacy following AAV treatment.

## 2. Materials and Methods

### 2.1. Animal Care and Neonatal AAV1-GALC Particle Injection

A protocol to execute the current study was approved by the Mayo Clinic Institutional Animal Care and Use Committee under protocol number A23106. Animal care and handling procedures were in compliance with the Guide for the Care and Use of Laboratory Animals [24]. A previous study showed that AAV1 administered to the brain via intraventricular injection in neonatal mice leads to robust and widespread expression of the lysosomal b-glucuronidase enzyme in the brain [25]. Based on this finding, AAV1-GALC was unilaterally injected into the lateral ventricle of pups from breeding pairs of heterozygous C57BL/6 *GALC*^Twi^ mice on PNDs 0 to 3 (6.0 × 10^10^ particles/dose). Before injection, pups assigned to the AAV1-GALC group were cryoanesthetized on wet ice for 10 min or until no movement was observed. Two microliters of AAV1-GALC solution was injected into the right lateral ventricle using a Hamilton syringe and a 30-gauge needle, as described previously [26]. After injection, pups were allowed to recover on a warm pad and returned to the cage with their mothers. Mice in the study, including untreated controls, were monitored daily until moribund. The control and the AAV1-GALC mice on a *GALC*^Twi/Twi^ background were identified by genotyping before PND 10 [9]. 

### 2.2. Construction and Production of AAV1-GALC Viral Particles

Murine *GALC* cDNA was subcloned into a pAAV2 vector under the control of a chicken β-actin promoter [27]. The pAAV2-GALC plasmid was then co-transfected with AAV helper plasmids, pDP1rs, into HEK293T cells to produce mouse GALC-expressing viral particles packaged in AAV serotype 1 capsid [26]. The transfected cells were lysed in the presence of 0.5% sodium deoxycholate and 50 U/mL benzonase by freeze and thaw cycles. The virus was isolated using a discontinuous iodixanol gradient and then purified on a HiTrap HQ column (GE Healthcare, Chicago, IL, USA). Upon elution, samples were buffer-exchanged to PBS using an Amicon^®^ Ultra 100 Centrifugation device (MilliporeSigma, Burlington, MA, USA). The genomic titer of the viral particle was determined by quantitative PCR using the ABI 7900 system (Thermo Fisher Scientific, Waltham, MA, USA). Briefly, genomic DNA was extracted by a standard method using DNase and Proteinase K to remove contaminated plasmids carried from transfection and decoat the viral particles, respectively. A standard curve was prepared by diluting the packaged plasmid to a range of 1E4 to 1E7 molecules per microliter. Primers (forward: 5′ GGCTGTTGGGCACTGACAAT 3′; reverse: 5′ CCGAAGGGACGTAGCAGAAG) were designed to target the woodchuck post-transcriptional regulatory element (WPRE) region of AAV. Therefore, primers exclusively detected viral particles with packaged DNA, but not empty particles. The PCR efficiency of the primer set was determined at 104% (slope = −3.2). Primer sets should have slopes of −3.0 to −3.6, which is 80–120% efficiency to be qualified for the application. Genomic DNA samples were diluted empirically to be in the range of the standard curve. Both the standards and samples were run in triplicate reactions by mixing 4 µL of DNA (samples or standards) and 8 µL of SYBR green master mix (Thermo Fisher Scientific, Waltham, MA, USA) per well. The ABI program for PCR was set at 95 °C for 10 min and then cycled 40 times (95 °C for 1 min and 60 °C for 30 s). Data were analyzed via the quantification software of the system. The titer of the AAV1-GALC particle was determined to be 3.25 × 10^13^ genomes/mL. The same batch of viral particles was used throughout the current study.

### 2.3. Determination of Psychosine Levels

Brain psychosine levels in the twitcher mice were always determined at the moribund stage. A group of wildtype mice at PND 41 (age-match to untreated twitcher mice) were included as controls for non-pathological psychosine levels. Hemibrains were divided into anterior- and posterior brain sections for psychosine analysis, such that the posterior portion contained midbrain, cerebellum, and brain stem. Brain samples were spiked with an internal standard, N,N-Dimethyl-D-erythrosphingosine (30 ng/mL), and then homogenized in 250 µL trifluoroacetic acid (5% *v*/*v*) in distilled water in a round-bottomed glass tube. We confirmed that there was no detectable levels of endogenous N,N-Dimethyl-D-erythrosphingosine in any of the brain samples. The homogenate was resuspended to 1 mL in 1:2 (volume-to-volume) chloroform/methanol with 5% trifluoroacetic acid and then centrifuged at 14,000 rpm for 5 min at 4 °C. The supernatant was carefully removed and transferred to a 2 mL centrifuge tube and then blown to dryness under a nitrogen stream. Samples were resuspended in 500 µL of an 80:20 (*v*/*v*) methanol/water solution with 0.1% formic acid and transferred to a clean sealed vial. Samples were analyzed with an API 365 LC/MS/MS triple quadrupole mass spectrometer (Thermo Fisher Scientific, Waltham, MA, USA).

### 2.4. Survivability and Body Weight Analysis

The body weights of the wildtype control mice (W04−07; Table 1) and AAV1-GALC treated (A01−A08; Table 1) and untreated (C01−C05; Table 1) twitcher mice were tracked and recorded weekly beginning at PND 10. There was no significant difference in body weight among groups at PND 10 (one-way ANOVA). The survivability of twitcher mice was determined as the natural lifespan without force feeding or other substantial interventions. Due to the progressive development of the disease phenotype in twitcher mice, animals were monitored daily to determine if the subject reached the moribund status, which was defined by complete inability to ambulate and/or feed. The mice were then sacrificed and harvested for analysis.

### 2.5. Immunohistochemistry

Mice were sacrificed by CO_2_ asphyxiation followed by perfusion with PBS (pH 7.4). Brains were rapidly dissected and cut into hemibrains for analysis. Hemibrains were immersion-fixed in neutral buffered 10% formalin for about 24 h at room temperature and then processed for paraffin embedding using an autoprocessor. Paraffin-embedded serial sections were cut in a sagittal orientation at 5 microns. Immunohistochemical staining of GALC and GFAP on paraffin-embedded brain sections was performed as described previously [15]. Briefly, paraffin serial sections were deparaffinized and rehydrated in xylene and a graded series of alcohol (100%, 100%, 95%, and 70%). Antigen retrieval was performed in double distilled water in a steam bath for 30 min. The sections were subsequently cooled to room temperature. The Dako Autostainer (Carpinteria, CA, USA) was used with the Dako EnVision HRP system for staining with a monoclonal anti-GALC antibody (CL13.1, 1:3000) or anti-GFAP antibody (GA-5; mouse monoclonal; 1:2500; BioGenex, Fremont, CA, USA) diluted in Dako Antibody Diluent with background-reducing components. A Dako Liquid DAB Substrate-Chromogen system was used as the chromogen. The slides were counterstained with hematoxylin (Thermo-Shandon, Pittsburgh, PA, USA) and subsequently dehydrated and coverslipped. Analysis was performed with light microscopy.

### 2.6. Luxol-Fast Blue (LFB)/Periodic Acid Schiff (PAS) Staining

Paraffin-embedded sections were stained according to a standard published protocol [28].

### 2.7. Liver GALC Analysis

A small piece of liver was homogenized in M-Per lysis buffer (Thermo Fisher Scientific, Waltham, MA) supplemented with protease inhibitor cocktail at a weight-to-volume ratio of 1:9. Total homogenate was centrifuged at 16,000× *g* for 1 h to obtain the detergent soluble fraction (supernatant). All samples were normalized to a 5 mg/mL total protein concentration. Forty microliters of the liver supernatant were incubated on a Maxisorp plate (Thermo Fisher Scientific, Waltham, MA, USA) pre-coated with an anti-mouse GALC antibody (CL1021) for 16 h at 4 °C. The well captured with GALC protein was then washed 2 times with PBS followed by GALC activity assay. Fifty-microliter reactions were set up containing 35 µL homogenate, 5 µL assay buffer (0.5 M sodium phosphate and 1 M sodium citrate; pH 4), 5 µL taurocholate-oleic acid mixture (20 mg/mL oleic acid and 70 mg/mL sodium taurocholate), and 5 µL of a 5 mg/mL solution of 2-hexadecanoylamino-4-nitrophenyl-β-D-galactopyranoside. The reaction mixture was incubated for 16 h at 37 °C. After incubation, 100 µL of a stop solution (0.1 M glycine and 0.1 M NaOH; pH 10.5) was added, followed by 200 µL absolute ethanol. The reaction mixture was vortexed and centrifuged for 10 min at 20,000× *g*. The absorbance of the supernatant was read at 410 nm.

### 2.8. Statistical Analysis

Statistical comparison between 2 groups was performed by the unpaired *t*-test at a 95% confidence interval. Determination of correlation was performed by Pearson correlation analysis (* *p* < 0.05; ** *p* < 0.01; *** *p* < 0.001; **** *p* < 0.0001).

## 3. Results and Discussion

The twitcher mouse is a naturally occurring model of infantile KD that exhibits a complete loss of GALC function [9]. Therefore, substrate accumulation in the CNS of this model happens early, and animals are onset with disease symptoms around PND 10. We hypothesized that performing AAV1-GALC gene replacement at neonatal stage would induce global CNS expression of GALC leading to substrate reduction. Given that psychosine levels are only mildly elevated on PND 1 in twitcher mice compared to wildtype controls [29], GALC expression at an early age could slow psychosine accumulation and alleviate disease progression. To replace the function of endogenous GALC, we constructed a murine *GALC* cDNA expression vector under the control of a chicken β-actin promoter on the AAV2 genomic DNA backbone, packaged in the AAV1 capsid. Gene replacement was achieved by injecting 6.5 × 10^10^ genomes of AAV1-GALC particles into the right ventricle of neonatal twitcher pups (*n* = 8) on or before PND 3. Control animals, including untreated twitcher mice (*n* = 5) and wildtypes, were housed, handled and studied alongside the treated mice until the moribund stage (Table 1). 

**Figure 1:**
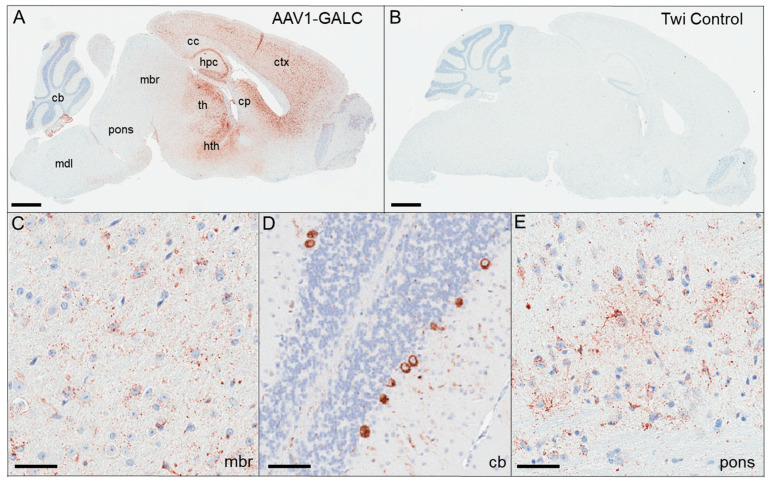
GALC protein distributions in control and AAV1-GALC-treated twitcher mice. GALC protein was analyzed by immunohistochemistry (IHC) using an anti-GALC antibody on a sagittal brain section of an AAV1-GALC-treated mouse (**A**) and an untreated twitcher mouse (**B**) harvested at moribund. The whole brain section is shown at a 4× magnification side-by-side for comparisons. GALC expression in the midbrain (**C**), the cerebellum (**D**), and the brain stem pons (**E**) is shown at a 20× magnification. Scale bars: 1 mm (**A**,**B**) and 50 µm (**C**–**E**). Abbreviation: cc—corpus callosum; ctx—cerebral cortex; hpc—hippocampus; cp—caudate putamen; th—thalamus; hth—hypothalamus; mbr—midbrain; cb—cerebellum; mdl—medulla.

To examine the brain expression of GALC protein from transgene, we performed immunohistochemistry (IHC) on sagittal brain sections of AAV1-GALC-treated mice. In our study, all the treated mice had a similar spatial distribution of GALC protein; therefore, GALC signals from a representative mouse (A02) were illustrated in Figure 1. We detected a widespread CNS distribution of GALC in the AAV1-GALC treated mice at PND 88 (Figure 1A), compared to an undetectable IHC-GALC signal in a control twitcher mouse (C02) at PND 42 (Figure 1B). GALC protein was predominantly expressed in the forebrain (Figure 1A), including the cerebral cortex (ctx), the corpus collosum (cc), the hippocampus (hpc), the thalamus (th), the hypothalamus (hth), and the caudate putamen (cp). At lower levels, GALC was also expressed in the midbrain (mbr) (Figure 1C), the cerebellum (Figure 1D), and the pons (Figure 1E). Distinctive patterns of neuronal expression were detected in hpc (Figure 1A), dentate gyrus (Figure 1A), and Purkinje cells (Figure 1D), which were less apparent in the medulla (mdl) (Figure 1A). 

To determine whether AAV1-GALC injected directly into brain ventricle was transduced into peripheral tissues, we examined GALC levels in liver tissue of the treated mice. No significant increase of GALC was detected in the treated mice compared with that in the control. Additionally, we tried examining GALC levels in the plasma of treated mice. However, we were not able to sample clear plasma from the twitcher mice at the moribund stage due to their severe dehydration state. Our results suggest that there was not significant, or at least detectable, leakage of AAV1-GALC into the peripheral tissues of animals in our study. 

**Figure 2:**
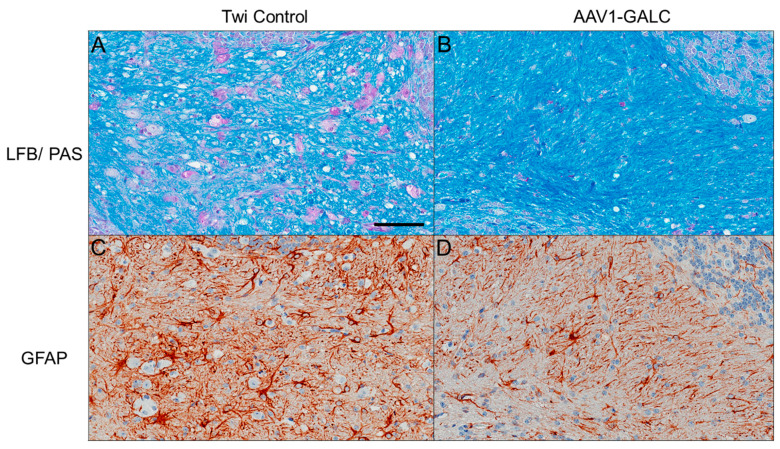
Alleviation of KD pathology in the cerebellar white matter region of an AAV1-GALC-treated twitcher mouse. Myelination and globoid cells were detected by LFB/PAS staining in an untreated twitcher mouse control (**A**) and an AAV1-GALC-treated mouse (**B**) harvested at the moribund stage. Astrogliosis levels were detected by GFAP IHC in the untreated control (**C**) and the AAV1-GALC-treated mouse (**D**). Scale bar: 100 µm (**A**) applied to all panels.

Pathological analysis of postmortem KD brains reveals extensive neuroinflammation, prominent astrogliosis (in white matter), and the presence of globoid cells, multinucleated macrophages characterized by the accumulation of lipids [30,31,32]. Looking at these pathologies in our AAV1-GALC-treated mice, which had widespread GALC expression, we observed that the number of globoid cells was dramatically reduced in the cerebellum white matter (cbwm). Additionally, the appearance of vacuoles detected in the cbwm, which is an indication of intramyelinic oedema, was also lowered in the AAV1-GALC-treated mice, as analyzed by LFB/PAS staining (Figure 2A vs. Figure 2B). Activated reactive astrocytes had elevated glial fibrillary acidic protein (GFAP) levels that were positively correlated with the extent of astrogliosis. An apparent reduction of GFAP signal was detected in the AAV1-GALC-treated mouse, which suggests an alleviation of astrogliosis compared to that in the control animals at the moribund stage (Figure 2C vs. Figure 2D).

**Figure 3:**
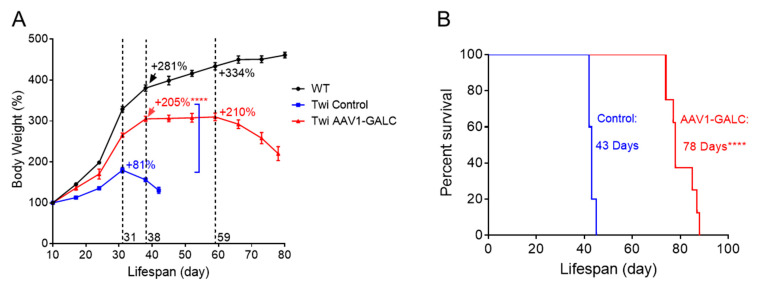
AAV1-GALC treatment efficacy on body weight gain and survivability. (**A**) Body weight (wildtype, untreated twitcher, and AAV1-GALC treated twitcher mice) tracked weekly from PND 10 to PND 80. The weight value is represented as a percentage relative to body weight at PND 10. (**B**) Survival plots of the untreated twitcher control (*n* = 5) and the AAV1-GALC-treated twitcher mice (*n* = 8). The median survival day of each group is annotated. Statistical analysis was performed by the unpaired *t*-test at 95% confidence levels (**** *p* < 0.0001).

In the current study, untreated twitcher mice gained weight until PND 31, followed by sharp deterioration over the next 10 to 12 days. The average maximal body weight attained was 181% of weight on PND 10, demonstrating a failure-to-thrive phenotype. In contrast, the body weights of twitcher mice treated with AAV1-GALC were partially rescued; the treated mice gained 205% of their body weight (**** *p* < 0.0001 vs. controls) from PND 10 to PND 38, compared to unaffected wildtype mice that gained 281% of their body weight over the same period. Moreover, the treated mice maintained their weights longer, up to PND 59, and then gradually deteriorated over the last three–four weeks of their lifespans (Figure 3A). The median lifespan of the AAV1-GALC treated mice was 78 days compared with that of the untreated controls of 43 days—an 81% increase (Figure 3B; **** *p* < 0.0001).

**Figure 4:**
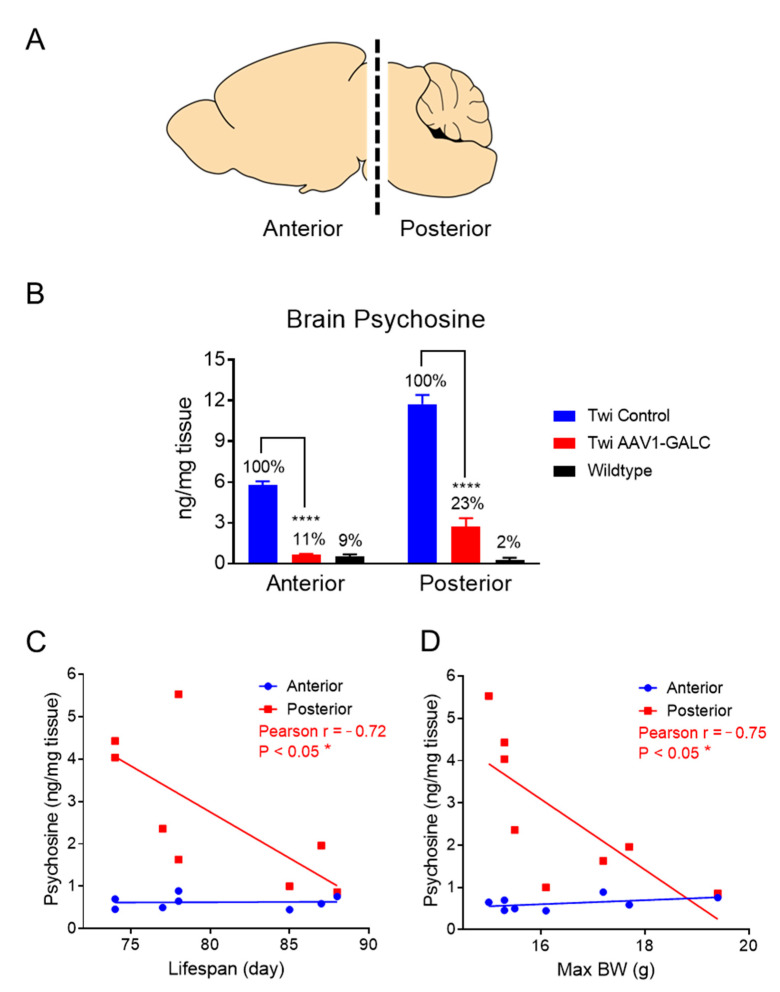
Brain psychosine clearance in AAV1-GALC-treated twitcher mice. (**A**) Schematic diagram showing the anterior and posterior brain portions analyzed for psychosine analysis. (**B**) Psychosine levels of untreated twitcher mice (blue), AAV1-GALC treated twitcher mice (red), and wildtype mice (black) in the anterior and posterior brains. Statistical comparison was performed by the unpaired t-test, **** *p* < 0.0001. (**C**) Correlation analysis between anterior (blue) or posterior (red) brain psychosine levels and the lifespan of AAV1-GALC-treated mice. (**D**) Correlation analysis between anterior (blue) or posterior (red) brain psychosine levels and the maximum body weight of AAV1-GALC-treated mice. Correlation analysis was performed by the Pearson correlation test, * *p* < 0.05.

Next, we analyzed the effect of *GALC* gene replacement on substrate clearance. Hemibrain divided into anterior- and posterior-brain portions was analyzed for the distribution of psychosine levels (Figure 4A). The anterior portion contained forebrain regions closest to the viral particle injection site (i.e., lateral ventricle); while the posterior portion contained the midbrain, the cerebellum, and brain stem regions, which were further away from the injection site. Psychosine is enriched in myelin, and a higher content of white matter is present in the posterior portion; thus, more psychosine accumulation takes place in these regions. Accordingly, psychosine levels were two times higher in the posterior brain compared to in the anterior brain in untreated animals (blue bars in Figure 4B; 11.8 vs. 5.8 ng/mg). AAV1-GALC treatment normalized psychosine levels in twitcher mice to a level indistinguishable from the wildtype in the anterior brain. Psychosine levels were reduced by 89% in the treated group compared to in the untreated group at the moribund stage (Figure 4B (left); **** *p* < 0.0001), which suggests that *GALC* gene delivery was highly efficient in regions proximal to the injection site. By comparison, AAV1-GALC administration resulted in a 77% (range: 53–92%) reduction of psychosine accumulation in the posterior brain of treated mice compared to in the posterior brain of the untreated mice at the moribund stage (Figure 4B (right); **** *p* < 0.0001). Given that cerebellum and brain stem are major pathological sites in KD and incomplete psychosine clearance was observed in these brain regions, we hypothesized that the degree of substrate clearance in the posterior brain may be associated with the severity of disease phenotype. Therefore, we performed a correlation analysis to determine whether psychosine levels in the posterior brain correlated to treatment efficacy. Interestingly, psychosine levels in the posterior brain, but not the anterior brain, were significantly and inversely correlated with the lifespan (Pearson r = −0.72, * *p* < 0.05; Figure 4C) and the maximum body weight (Pearson r = −0.75, * *p* < 0.05; Figure 4D) of the AAV1-GALC-treated mice. Our results suggest that following viral particle injection to the lateral ventricle, the level of GALC activity achieved in remote brain regions is not sufficient to attain complete substrate clearance and thereby the maximum treatment efficacy.

AAV1 vector-mediated gene therapy has been used in several human clinical studies, including the treatment of lipoprotein lipase deficiency [33], antitrypsin deficiency [34], and muscular dystrophy [35]. It has also been shown to yield robust transgene expression in the CNS of several animal models [36,37,38,39]. In light of these recent studies, we chose the AAV serotype 1 vector for our study. To maximize the CNS distribution of viral vector infection, we administered AAV1-GALC via injection directly into the lateral ventricle during the neonatal period. Our intent was that the viral vector would enter the cerebrospinal fluid (CSF) flow and be delivered globally in the CNS through the ventricular system [40]. Compared to one previous study using the same AAV1 serotype [41], we achieved more widespread expression of GALC in the CNS and, therefore, observed a more significant correction to lifespan extension (i.e., medians of 78 vs. 55 days). Since both studies used strong constitutive promoters and murine *GALC* cDNA, the difference in efficacy may likely be a result of the higher AAV1-GALC dose administered in the current study (i.e., 6.5 × 10^10^ vs. 3 × 10^10^ genomes). A more recent study that injected 1 × 10^11^ genomes of AAVhu68-human GALC to the lateral ventricle of twitcher mice achieved an improvement in the median lifespan of 130 days [42]. Differences in AAV serotype and dosage likely account for observed differences in survivability. To our knowledge, our work represents one of the few preclinical studies with AAV-GALC gene therapy that highlights the correlations between psychosine clearance and treatment efficacy.

Among existing studies in twitcher mouse-related models [41,42,43,44,45,46,47], murine GALC expressed by AAV was used to evaluate treatment efficacy in all, but one study that used human GALC, as mentioned above [42]. While there are no dramatic structural differences between murine and human GALC, the protein sequence identity between the two species is determined at only 83% (based on NCBI blastp suite-2sequences alignment; NP_000144 vs. EDL18937). Therefore, key residues known to mediate interactions for transportation through trafficking machinery [48,49] and for co-activation through saposin A [50] may be varied between species and may therefore impact human GALC function in animal models. For example, murine residue serine-146 forms a hydrogen bond with residue lysine-19 on murine saposin A [50]. However, the same position on human GALC is an aspartic acid, which has a longer carbon chain backbone, and therefore may alter the hydrogen bond formation, the GALC−saposin A interaction, and GALC activity. Additionally, expression of human GALC may trigger a host immune response that neutralizes the function of therapeutic protein in mouse models with endogenous mutant GALC expression such as the Twi-5J mouse [51] and the CRISPR-Cas9 human mutation knock-in models [52]. Taken together, these examples explain the tendency toward using murine GALC for AAV gene therapy studies in mouse models. 

AAV-GALC gene replacement studies in twitcher mice have also yielded mixed results with varied efficacy. Interestingly, a strategy for AAV-GALC delivery targeted to the cerebellum led to only minor clinical improvements, which suggests that GALC replacement in forebrain is crucial for increased efficacy [53]. To date, the AAV-GALC study that achieved the highest level of efficacy utilized AAV serotype rh10 that combined intracerebroventricular and intracerebellar administrations together with repeated intravenous injections to target peripheral tissues. That strategy allowed 25% of treated twitcher mice to live up to eight months [45] and highlights the importance of GALC replacement into peripheral tissues. Numerous AAV-GALC gene therapy studies using various administration routes, AAV serotypes, and animal models have been well reviewed recently [54,55,56].

## 4. Conclusions

Overall, AAV1-GALC gene replacement by unilateral intracerebroventricular administration at the neonatal age led to widespread expression of functional GALC protein in the CNS, which was associated with alleviation in neuropathology, psychosine clearance, and slower disease progression in the twitcher mouse model of KD. 

## Figures and Tables

**Table 1:** Summary of experimental groups, lifespan, brain psychosine levels, and the maximum body weight gain.

ID	Genotype	Exp Group	Treatment Age (PND)	Lifespan (PND) Median (%)	Psychosine (ng/mg) Mean (%)		Max BW (% PND10)
					Anterior	Posterior	
C01	Twi	Control		45	6.2	13.8	179
C02	Twi	Control		42	6.0	11.5	204
C03	Twi	Control		42	5.6	9.6	188
C04	Twi	Control		43	4.7	11.5	165
C05	Twi	Control		43	6.3	12.3	167
				43 (100%)	5.8 (100%)	11.8 (100%)	181
A01	Twi	AAV1-GALC	0	78	0.9	1.6	331
A02	Twi	AAV1-GALC	0	88	0.8	0.9	366
A03	Twi	AAV1-GALC	0	77	0.5	2.4	304
A04	Twi	AAV1-GALC	0	87	0.6	2.0	322
A05	Twi	AAV1-GALC	3	74	0.7	4.4	319
A06	Twi	AAV1-GALC	3	74	0.5	4.0	306
A07	Twi	AAV1-GALC	1	78	0.7	5.5	288
A08	Twi	AAV1-GALC	1	85	0.5	1.0	298
				78 (181%)	0.6 (11%)	2.7 (23%)	317
**Controls for psychosine analysis**							
W01	WT	Control		41	0.8	0.1	
W02	WT	Control		41	0.4	0.0	
W03	WT	Control		41	0.4	0.6	
					0.5 (9%)	0.3 (2%)	
**Controls for BW analysis**							
W04	WT	Control		100			475
W05	WT	Control		100			495
W06	WT	Control		100			521
W07	WT	Control		100			500
							498

(%): percentage relative to the median or mean of the twitcher control group. Abbreviations: Twi—twitcher; WT—wildtype; Exp—experimental; PND—postnatal day; Max BW—maximum body weight.

## Data Availability

The data that support the findings of this study are all included in the manuscript.
